# Impact of Observed and Controlled Water Intake on Reticulorumen Temperature in Lactating Dairy Cattle

**DOI:** 10.3390/ani8110194

**Published:** 2018-10-31

**Authors:** Melissa C. Cantor, Joao H. C. Costa, Jeffrey M. Bewley

**Affiliations:** 1 Department of Animal and Food Sciences, University of Kentucky, Lexington, KY 40546, USA; costa@uky.edu; 2 Cow Focused Housing, Bardstown, KY 40004, USA; jmbewley@gmail.com

**Keywords:** temperature, reticulorumen, water intake, dairy cattle, precision dairy technology

## Abstract

**Simple Summary:**

Precision technologies are often integrated on dairy farms to monitor individual animal health. One precision technology used is a bolus that is inserted into a cows’ reticulorumen to monitor reticulorumen temperature. Although it is known that both water temperature and water quantity ingested are associated with changes in reticulorumen temperature, limited quantifiable research exists on the impact and magnitude of controlled water intake on reticulorumen temperature. We conducted an observational study to determine the effect of natural water ingestion on reticulorumen temperature, and performed a modified Latin square where cows were drenched with specific water quantities at specific temperatures to determine the effect of controlled water intake on reticuloruminal temperature. Our results demonstrated that water quantity and water temperature affect not only the magnitude of change in degrees in Celsius, but also how much time is required for reticulorumen temperature to return to baseline. This study provides insights in how to adjust the temperatures measures affected by water intake when using cattle reticulorumen temperature monitoring systems and how to estimate water consumption using decreases in reticulorumen temperature.

**Abstract:**

Dairy precision technologies helps producers monitor individual animals. Reticulorumen temperature boluses are a way to monitor core body temperature; however, factors such as water intake affects reticulorumen temperature. This research determined the effect of natural water intake and a controlled water drench on reticulorumen temperature (RT) in dairy cattle. In observational study part 1, tie- stall cows (*n* = 4) with RT transponders were observed for natural water intake (recorded by in line water meters) for 48 h. In experiment part 2, a randomized Latin square design with cows (*n* = 12) restricted on feed for 4 h, were drenched daily with a water quantity of 6.7 L, 11.4 L or 22.7 L, and at controlled water temperature of 1.7 °C, 7.2 °C, 15.5 °C, or 29.4 °C. Descriptively, observational study 1 had (Mean ± SD 0.27 ± 0.31 L ingested per drinking event (*n* = 84) and RT decline from baseline was 2.29 ± 1.82 °C. For the experiment, a 48-h specific rolling baseline temperature range (BTR) was calculated for each cow prior to the experiment to determine time required for RT to reach BTR, and time to return to BTR. In part 2 of the experiment, as water quantity increased, RT had a greater maximum degree drop from baseline. Water temperature and water quantity interaction influenced time required for BTR to reestablish. The coldest water temperature at the highest drench quantity affected time for BTR to reestablish the longest (103 min). Results from this study suggest that an algorithm could be designed to predict water intake events for producers using reticulorumen temperature.

## 1. Introduction

Preventative health measures may reduce the impact of animal disease and improve animal health, cow longevity, and milk production [1,2,3]. One way to intervene prior to the emergence of clinical symptoms of disease is by utilizing constant measures to detect slight deviations, such as using a reticulorumen temperature transponder inserted into a cow’s reticulorumen to detect changes in core body temperature. Temperature monitoring technologies such as a reticuloruminal temperature transponders take multiple temperature measurements [4], providing the precision of identifying deviations from an individual cow’s temperature and circadian rhythm [5]. Additionally, reticuloruminal temperature transponders can detect fever without investing in labor required to take a rectal measurement. However, there are two potential limitations to monitoring dairy cattle temperature with reticuloruminal temperature transponders. First, dry matter intake is positively correlated to reticuloruminal temperature, as seen in finisher cattle fed a high concentrate diet [6]. Indeed, dairy cattle fed higher concentrate to forage ratio diets compared to moderate concentrate to forage ratio diets had higher reticuloruminal temperatures associated with sub-acute acidosis [4]. However, sub-acute ruminal acidosis is a metabolic disorder in cattle, and fever can still be detected under sub-acute ruminal acidosis conditions [4]. Therefore, while feed intake does influence reticuloruminal temperature, a balanced total mixed ration diet fed to dairy cattle with adequate forage should not induce such pronounced effects on temperature within the reticulorumen. Another potential limitation of monitoring dairy cattle temperature with temperature transponders placed in the reticulorumen is the effects of water intake on reticulorumen temperature.

The relationship between water intake and dairy cattle health has previously been investigated suggesting that monitoring water intake could be used as a tool for reducing morbidity rates [7]. In addition, high producing Holsteins experience higher increases in reticulorumen temperature during summer than lower producing cattle, suggesting an increased sensitivity to heat stress [8]. However, several studies demonstrated that water intake significantly decreased reticuloruminal temperature for extended periods [9,10,11]. Both water quantity and water temperature affect reticulorumen temperature [9,11,12]. The variation in time required to return to basal reticuloruminal temperature is broad among previous studies ranging from 20 min to over 3 h [11,12,13]. Feed and water intake both affect temperature within the reticulorumen and may explain such a large variation in time required for return to basal levels [4].

Therefore, it is of benefit to quantify both the observational effects of natural water intake on reticulorumen temperature, and the controlled effects of water quantity and water temperature on the reticulorumen. It was previously reported that water intake for dairy cows housed in tie-stall environments occurred in smaller, more frequent drinking events estimated around 2 L, though this study paired cows to drinking cups, making individual drinking events unknown; in contrast, freestalls environments had drinking events less frequent (7 times per day), but higher in volume (13 L per visit) [7,14]. Therefore, to our knowledge, no study has assessed the influence of water intake on reticulorumen temperature in dairy cattle in (1) a tie-stall setting, or (2) determined the influence of controlled water quantity and water temperature on reticulorumen temperatures. Monitoring for these temperature drops demonstrates the potential for monitoring water intake, and further understanding of the influence of water intake on reticulorumen temperature. The objective of observational study part 1 was to determine in a research setting, if small quantities of water ingested had an effect on reticuloruminal temperature. The objective for experiment part 2 was to define the impact of water intake on reticulorumen temperatures in water intake scenarios.

## 2. Materials and Methods

Animal ethics approval was provided by the University of Kentucky’s Institutional Animal Care and Use Committee (IACUC) before enrollment on these studies. All personnel were trained and animals were deemed healthy and in condition for inclusion before they participated in the study. The study was conducted in accordance with the Declaration of Helsinki, and the protocol was approved by the Ethics committee of the University of Kentucky protocol number: 2010-0761.

In observational study part 1 (study 1), four mid-lactation (159 ± 39 days in milk (DIM)), multiparous (2.5 ± 0.5 lactations), Holstein dairy cows producing in average 36.7 ± 2.5 kg of milk daily were equipped with reticulorumen bolus transponders (SmartBolus^®^, TenXSys, Eagle, ID, USA) set to record reticulorumen temperature at 2-min intervals. Mean somatic cell count (SCC) was 125,000 ± 26,575 SCC/mL at the most recent dairy herd improvement test prior to the experiment beginning. All cows were confirmed pregnant one week before study 1 via transrectal ultrasonography scan of the uterus. Cows were originally housed in a free stall barn at the University of Kentucky Coldstream Dairy Research Farm and were provided a five-day habituation period to adjust to the tie-stall barn before intensive observation on 24 and 25 January 2011. The mean daily low and high ambient temperatures during study 1 were −9.44 °C and 2.78 °C, respectively (Bluegrass Airport, Latitude 38.04, Longitude-84.60). Study 1 was conducted during winter to avoid heat stress and to control influence of increased external temperatures on the reticulorumen temperature. Cows were fed a total mixed ration (TMR) *ad libitum* formulated following the National Research Council (NRC) guidelines to meet or exceed the requirements of lactating dairy cows producing at least 39 kg of milk daily (NRC, 2001). Total mixed ration (TMR) as percent of dry matter (DM) consisted of 25.8% corn silage, 15.0% grass silage, 2.7% alfalfa hay, 8.3% cottonseed and 48.2% concentrated mix (CPC Commodities, Fountain Run, KY, USA); the total mixed ration was on average 49.1 ± 1.5% dry matter. The TMR was provided 2 times per day at 05:30 and 14:00. Cows were milked 2 times per day at 04:00 and 15:30. Cows were removed from tie-stalls for milking before the rest of the herd and were not permitted water access during milking. One poly water bowl (SMB MFG, Wallenstein, ON, Canada) equipped with a range water meter (Recordall Badger Meter^®^, Badger Meter, Milwaukee, WI, USA) was assigned to each tie-stall. Four external reticulorumen bolus transponders submerged in the cows’ water bowls recorded water temperatures at 2 min intervals. Two observers monitored two cows each (*n* = 4) for natural drinking behavior for 48 consecutive hours. In order to prevent fatigue of observers, observers rotated observations in 8-h shifts, but at least 2 observers were watching cows drink water at any given time.

Mean (±SD) 24-h daily water consumption (L/drinking event) for all cattle were recorded. Because of frequent, small drinking events in the tie-stall environment, the termination of a drinking event was established when 30 min elapsed without another drink bout. The sum of all water consumed within each drinking event represented the water quantity consumed per event. In this manner, smaller, frequent drinking events were not included in analysis because the amount of time elapsed between each drink bout was not sufficient for temperatures to return to baseline before the next drink bout occurred. Thus, the water consumed in the drinking events examined in this study does not represent total daily water consumption. For this study, a cow-specific rolling baseline temperature range (BTR) was calculated using temperatures recorded within the most recent 30 min since drinking events were frequent. This BTR was used to define the first time when reticuloruminal temperature returned to baseline following a drinking event.

In experiment part 2 (experiment) twelve mid-lactation (160 ± 45 DIM), multiparous (2.5 ± 0.5 lactations), Holstein dairy cows producing in average 31.9 ± 8.7 kg of milk daily were equipped with reticulorumen bolus transponder set to record reticuloruminal temperature at 2-min intervals. In order to ensure that the experimental procedure (restricting feed and water intake for several hours) did not affect body temperature, an additional 4 negative control cows were randomly selected and enrolled. All control cows were negative controls and did not receive water drenching, but were subject to the same feed and water restriction, and housed in tie stalls. Rectal temperature using a digital thermometer (GLA M700, GLA Agricultural Electronics, San Luis Obispo, CA, USA) with a temperature range of 0 °C to 65 °C, 0.1 °C SD, was taken on all cows every 60 min in order to confirm that the drenching procedure did not affect rectal temperature. Mean SCC was 133,000 ± 24,550 SCC/mL at the most recent dairy herd improvement test. All cows were selected based on a healthy, (not lame, no metabolic disease) and confirmed pregnancy status one week before experimentation, via transrectal ultrasonography scan of the uterus. Cows were housed in a tie-stall barn at the University of Kentucky Coldstream Dairy Research Farm. The experiment was conducted daily from 11:00 until 15:30 from 16 to 19 February 2011. The mean daily low and high ambient temperatures were 2.78 °C and 4.44 °C, respectively (Bluegrass Airport LEX). In this experiment, water volume and temperature were controlled. Water temperature was controlled by use of a digital thermometer with a straight probe (GLA M700, GLA Agricultural Electronics, San Luis Obispo, CA, USA), refrigerator for colder temperatures (ABT-12S-TS, American Biotech Supply, Temecula, CA, USA) and water bath (Digital Water Bath, Shel Lab, Cornelius, OR, USA) range 5 °C to 80 °C, ±0.2% at 37 °C. Water was administered to each cow via drenching utilizing a cattle drench (Cattle Pump System^®^, Springer McGrath Co., Mc Cook, NE, USA) once daily four consecutive days using a modified Latin Square design (depicted in Table 1). The drench quantities chosen were selected to represent a small (5.7 L) drinking event for a cow housed in a free stall environment, an average drinking event (11.4 L) for a cow based on the literature [13], and the large quantity (22.7 L) of water was selected to determine the effect of a water quantity, water temperature interaction on degrees dropped from BTR and time required to return to baseline. 

All random selections were conducted using a random number table. Each of the twelve cows received a constant water quantity of 5.7 L, 11.4 L, or 22.7 L once daily each of the four days. Water temperatures were randomly assigned at 1.7 °C, 7.2 °C, 15.6 °C, and 29.4 °C, such that no cow received the same temperature treatment twice.

Feed and water intake were restricted daily from 8:30 until release for the afternoon milking at 15:30. Prior to feed restriction, cows were offered the same TMR as study 1. Time of water treatment administration (beginning and end) was recorded for each cow. For the experiment, since feed intake and water intake were restricted, a cow-specific baseline temperature range (BTR) was calculated using the reticuloruminal temperatures recorded 48 h before the beginning of the experiment. This BTR was used to define the time when reticuloruminal temperature returned to baseline following water drenching. All statistical analysis procedures were performed in SAS^®^ (SAS, Cary, NC, USA) and statistical significance was considered at (*p* < 0.05). Pearson’s correlations (PROC CORR) were performed between rectal temperatures and reticuloruminal temperature on the hour prior to drenching, and 1 h after drench for the cows subject to experimental procedure. Pearson’s correlations were also performed separately for the 4 negative control cow’s rectal temperature 1 h after the experimental cows received drenching treatment. The MIXED procedure was used to assess the effect of water quantity (i.e., 5.7 L, 11.4 L, 22.7 L), water temperature (i.e., 1.7 °C, 7.2 °C, 15.6 °C and 29.4 °C) and their interaction on the maximum degrees (°C) dropped in reticuloruminal temperature. Cow was a fixed effect and block by water quantity interaction were random effects. The overall line equation for this model was used to explain the maximum temperature drop.

A second mixed model was used was used to assess the effect of water quantity (5.7 L, 11.4 L, 22.7 L), water temperature (1.7 °C, 7.2 °C, 15.6 °C and 29.4 °C) and their interaction on the time (min) required for reticuloruminal temperature (°C) to return to baseline. Cow was a fixed effect and block water quantity interaction were random effects. In order to interpret the water quantity (L) water temperature (°C) interaction, the differences in (LSM ± SEM min) were also generated from the mixed model. Another overall line equation model was used to explain the time to return to baseline.

## 3. Results and Discussion

In study 1, the mean (±SD) 24 h daily water consumption for all mid-lactation dairy cattle was 38.15 ± 3.58 L. At the univariate level, there was no association between feed intake (kg/d) and ruminal temperature for the tie-stall cattle for study 1 (*p* = 0.35), which was likely a result of feed intake (43.8 ± 3.0 kg) between cows not being different. Therefore, a lack of differences in feed intake in our observational study was likely masking the ability to isolate the effects of feed intake on reticuloruminal temperature. We suggest that our dairy cattle had similar feed intakes because they were balanced by health status, stage of lactation, and confirmed pregnant. However, it is established that in dairy cattle with ad libitum access to water and feed, both a moderate concentrate diet and a high concentrate diet increases reticuloruminal temperature, though the higher concentrate diet elevated the reticuloruminal temperature for longer durations of time [4]. Therefore, both feed intake and gut fill affect reticuloruminal temperature, and the duration of temperature change is likely associated with rumen fermentation. Future directions should investigate the rumen fill and feed intake influence in combination with water intake’s influence on reticuloruminal temperature. Mean (± SD) volume of water consumed per drinking event and descriptive statistics are in (Table 2).

The mean water consumption per drinking event in study 1 was 0.5 L, and differed from the mean water consumption (2 L) suggested for tie-stall cattle [7]. This study part 1 drinking event intake is different than Lukas et al. (2008) because they approximated water intake since cattle were assigned to drinking bowls in pairs [7]. Since our study had individual cups assigned to cows, it is likely that the cattle in study 1 with their own drinking cups, ingested more frequent, smaller quantities of water when water sources are accessible within tie-stalls. Study part 1 suggests even small quantities of water intake influence reticuloruminal temperature for approximately an hour, though our interpretations are limited in this preliminary study. This provides preliminary information for research involving the reticuloruminal bolus, researchers should acknowledge even water access in the milking parlor may affect reticuloruminal temperature. Future research should investigate the effects of small water intakes and feed intake on reticuloruminal temperature in an experimental setting.

For the experiment, rectal temperatures were 38.0 ± 0.50 °C (mean ± SD) across the experimental period. Pearson’s correlations for rectal temperature and reticuloruminal temperature on the hour prior to drenching were strong (r = 0.72). For the 1 hour after drenching, Pearson’s correlations between rectal temperature and reticuloruminal temperature were weak (r = 0.27) for the experimental cows, but remained strong for the negative control cows (r = 0.72). These results suggest that water drenching was the cause of temperature changes within the reticulorumen. Our correlations prior to drenching agree with others who compared reticuloruminal bolus temperatures to rectal temperature in dairy cattle who had access to feed [15]. Our correlations for rectal temperature and reticuloruminal temperature after drenching also agree with others who drenched cattle restricted on feed and water [12]. This suggests that water intake affects reticuloruminal temperature.

**Figure 1:**
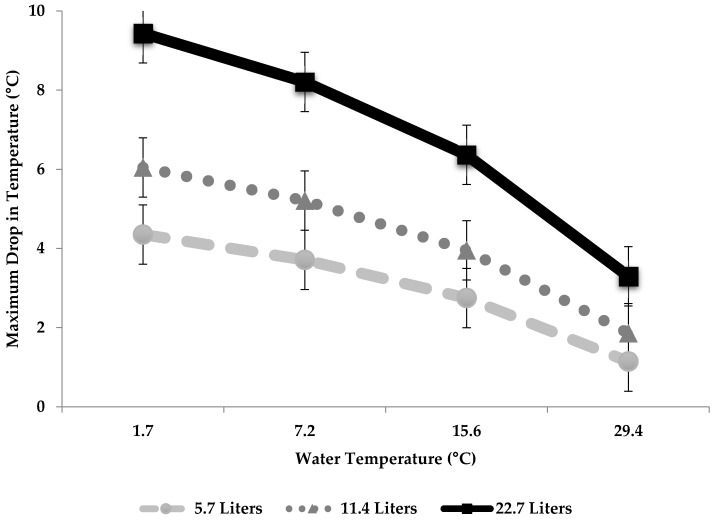
The effect of water quantity (5.7 L, 11.4 L, 22.7 L), water temperature (1.7 °C, 7.2 °C, 15.6 °C and 29.4 °C) and their interaction on the maximum degrees (°C) dropped in reticuloruminal temperature for feed restricted, once daily drenched pregnant cows (*n* = 12) explained with the equation: Temperature Decrease = 1.56 + (1.45 × Water Quantity (L)) − (0.003 × Water Temperature (°C)) − (0.04 × (Water Quantity (L) × Water Temperature (°C)).

For the experiment, water quantity independently affected maximum reticuloruminal degrees (°C) dropped, whereas water temperature (L), and the interaction were not significant (Table 3, depicted in Figure 1). The overall line equation for this model was: Temperature Decrease = 1.56 + (1.45 × Water Quantity (L)) − (0.003 × Water Temperature (°C)) − (0.04 × (Water Quantity (L) × Water Temperature (°C)).

The results of this study disagree with Bewley et al., 2008, where feed restricted cattle drenched with either (19 kg water) that was either cold 5 °C or hot 39 °C had effects of both water quantity and water temperature on maximum reticuloruminal degrees (°C) dropped [12]. Results likely differed in this study because of experimental design differences, as we used water temperatures varying across the temperature spectrum and Bewley et al. (2008) used temperature extremes [13]. The results of this study suggest that the quantity of water ingested by the cow likely affects the magnitude of reticuloruminal temperature change.

**Figure 2:**
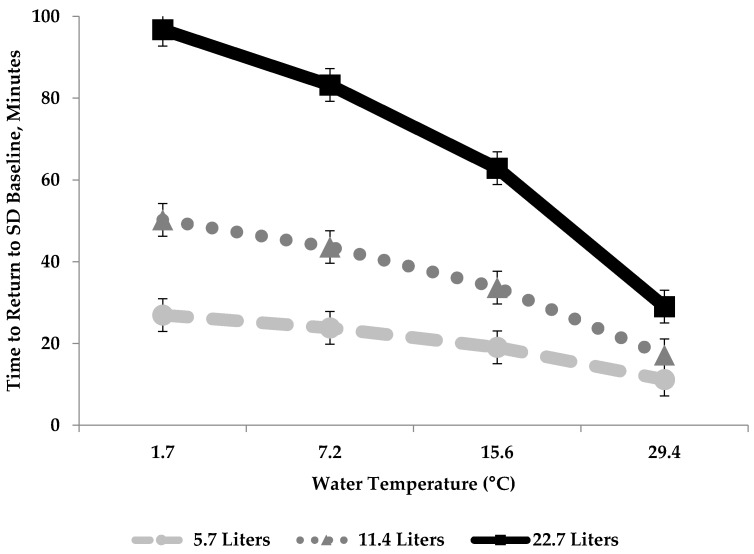
The effect of water quantity (5.7 L, 11.4 L, 22.7 L), water temperature (1.7 °C, 7.2 °C, 15.6 °C and 29.4 °C) and their interaction on the time (min) required to return to baseline reticulorumen temperature (°C) for feed restricted, once daily drenched pregnant cows (*n* = 12) explained with the equation: Time to Return to Baseline = 4.33 + (16.06 × Water Quantity (L)) − (0.01 × Water Temperature (°C)) − (0.40 × (Water Quantity (L) × Water Temperature (°C)).

For the experiment, water quantity (L), and the interaction, affected modeled time (min) for reticuloruminal degrees (°C) to return to baseline temperature (Table 4, depicted in Figure 2). Our results suggest that the time required to return to BTR is dependent on both water quantity and water temperature. Differences of LSM ± SEM min time to return to BTR for the water quantity water temperature interaction are reported in (Table 5). The explanatory equation for this model was found to be: (Time to Return to Baseline = 4.33 + (16.06 × Water Quantity (L)) − (0.01 × Water Temperature (°C)) − (0.40 × (Water Quantity (L) × Water Temperature (°C)).

To our knowledge, the interaction between water quantity and water temperature and its effect on time to return to BTR was not previously explored. This makes comparisons to the literature difficult. However, results from others, suggest that water quantity and water temperature may be dependent on one another, even if not explored in their models. For example, Brod et al. (1982) observed the coldest water drenches 1.7 °C affected reticuloruminal temperature for the longest time [14]. This is similar to our study, where the largest water quantity combined with the coldest water temperature affected the time to return to baseline the greatest within the reticulorumen. Conversely, smaller water quantities at warmer temperatures affected the time to return to baseline within the reticulorumen least. These observations are also seen in others; Bewley et al. (2008) observed that larger maximum reticuloruminal temperature drops (8.5 °C) affected return to reticuloruminal temperature baseline for a longer time [12]. Both observations in these studies suggest that water quantity’s influence on time to return to BTR within the reticulorumen is not independent of water temperature. However, these studies did not include the interaction within their models; therefore, we cannot make inferences, only speculations. However, based on our results of our experiment, we suggest the highest water quantity at the coldest water temperature affected the reticulorumen the longest, at 103 min, and the interaction which affected the reticulorumen least is less clear.

As herd size increases, more farms will convert to freestall environments where drinking events are observed as large and infrequent compared to tie-stall housed cattle [7,14]. Therefore, cows would likely ingest water quantities similar to this study’s drenching water quantity of 11.4 L, and colder water temperature interactions may affect reticuloruminal temperature for a greater time. For future research, ambient water temperature needs to be taken into consideration with water quantity when developing an algorithm to eliminate water drinking events. Ambient water temperature is likely warmer in the summer, and colder during the winter season. Future research should determine if ambient water temperature in freestall settings affects the time for return to BTR within the reticulorumen as seen in our experimental settings as cattle who produce more milk have higher reticuloruminal temperatures, and providing colder water may reduce heat stress [8]. Thus, reticuloruminal transponders hold much promise for monitoring water intake in dairy cattle and may provide a useful tool for detecting heat stress in high producing animals.

In summary, this study successfully quantified that tie-stall cattle drink water frequently in small water quantities. In an experimental setting, when feed intake is restricted, only water quantity affects the maximum degree drop within the reticulorumen. However, when considering the time to return to an individual cow’s specific rolling baseline temperature prior to water ingestion, water quantity is dependent on the ambient water temperature consumed. Higher quantities of water at a lower temperature affect an individual cow’s specific rolling baseline temperature for the greatest intervals. This research indicates reticuloruminal transponders hold the potential to successfully identify water intake noise. 

## 4. Conclusions

This research suggests that temperature monitoring via the reticulorumen is an effective means of monitoring a dairy herd for water intake; however, temperature readings must be examined with caution. The time required for reticulorumen temperature to return to baseline temperature is dependent upon water quantity and water temperature. Even small quantities of water affect reticuloruminal temperature in lactating dairy cattle. Colder water and greater quantities both affect the degree of change in reticulorumen temperature to the greatest magnitude. However, results from this study suggest an algorithm could be designed to predict water drinking events (bouts) for producers using reticulorumen temperature transponders. Future directions should develop an algorithm to potentially eliminate water drinking events in order to clean the data and look at reticuloruminal temperature related to rectal temperature. Future research directions should determine if reticulorumen temperature transponders can predict water intake in cattle during heat stress.

## Figures and Tables

**Table 1:** Modified Latin Square experimental treatment assignments (experiment part 2) for feed restricted, pregnant, mid-lactation cows (*n* = 12) receiving the same water quantity drench on each day at a different experimental water temperature.

Cow	Day 1	Day 2	Day 3	Day 4
**5.7 L of water drench**				
1	7.2 °C	29.4 °C	1.7 °C	15.6 °C
2	7.2 °C	1.7 °C	29.4 °C	15.6 °C
3	29.4 °C	15.6 °C	1.7 °C	7.2 °C
4	1.7 °C	7.2 °C	15.6 °C	29.4 °C
**11.4 L of water drench**				
5	15.6°C	29.4 °C	1.7 °C	7.2 °C
6	7.2 °C	1.7 °C	29.4 °C	15.6 °C
7	29.4 °C	15.6 °C	7.2 °C	1.7 °C
8	1.7 °C	7.2 °C	15.6 °C	29.4°C
**22.7 L of water drench**				
9	15.6 °C	29.4 °C	1.7 °C	7.2 °C
10	7.2 °C	1.7 °C	29.4 °C	15.6 °C
11	29.4 °C	15.6 °C	7.2 °C	1.7 °C
12	1.7 °C	7.2 °C	15.6 °C	29.4 °C

**Table 2:** Descriptive statistics for a consecutive 48-h observational study of 84 drinking events from 4 lactating, tie-stall housed Holstein cows with *ad libitum* feed and water access ^1,2,3^.

Parameter	Mean ± SD
Volume of water consumed per drinking event, L	0.27 ± 0.31
Water temperature before drinking event, °C	3.63 ± 3.14
Reticulorumen temperature before drinking event, °C	39.76 ± 0.49
Reticulorumen temperature decrease after drinking event, °C	2.29 ± 1.82
Time to return to baseline temperature, min ^2^	57.75 ± 38.70

^1^ The termination of a drinking event was established when thirty min elapsed without another drink. ^2^ A cow-specific rolling baseline temperature range (BTR) was calculated using the mean ± 3 SD of each temperature recorded within the most recent 30 min. This BTR was used to define the first time when reticuloruminal temperature returned to baseline following a drinking event. ^3^ Feed was provided as a total mixed ration at 05:30 and 14:00.

**Table 3:** Experiment part 2: Effect of water quantity (5.7 L, 11.4 L, 22.7 L), water temperature (1.7 °C, 7.2 °C, 15.6 °C and 29.4 °C) and their interaction using a linear mixed model investigating the maximum degrees (°C) dropped in reticuloruminal temperature for feed restricted, once daily drenched pregnant cows (*n* = 12).

Effect	Numerator DF	Denominator DF	LSM ± SEM	F Value	*p*-Value
Water quantity, L	1	9	2.85 ± 0.75	19.42	<0.01
Water temperature, °C	1	31	−0.08 ± 0.05	2.88	0.10
Water quantity, (L) × Water temperature, (°C)	1	31	−0.02 ± 0.01	4.17	0.05

**Table 4:** Experiment part 2: Effect of water quantity (5.7 L, 11.4 L, 22.7 L), water temperature (1.7 °C, 7.2 °C, 15.6 °C and 29.4 °C) and the interaction using a linear mixed model investigating the time (min) required after a reticuloruminal temperature drop to return to an individual cow’s baseline for experiment part 2 feed restricted, once daily drenched pregnant cows (*n* = 12).

Effect	Numerator DF	Denominator DF	LSM ± SEM	F Value	*p*-Value
Water quantity, L	1	9	23.60 ± 4.0	39.84	<0.01
Water temperature, °C	1	31	0.03 ± 0.24	0.02	0.90
Water quantity, (L) × Water temperature, (°C)	1	31	−0.23 ± 0.06	15.67	< 0.01

**Table 5:** The differences in (LSM ± SEM min) time for water quantity (5.7 L, 11.4 L, 22.7 L), water temperature (1.7 °C, 7.2 °C, 15.6 °C and 29.4 °C) interaction on time required to return to baseline reticulorumen temperature (°C) ^1^ for feed restricted, once daily drenched pregnant cows (*n* = 12) explained with the equation: Time to Return to Baseline = 4.33 + (16.06 × Water Quantity (L)) − (0.01 × Water Temperature (°C)) − (0.40 × (Water Quantity (L) × Water Temperature (°C)).

Water Quantity L	Water Temperature °C	LSM ± SEM (min)	*p*-Value
5.7	1.7	25.3 ± 11.0	0.01
5.7	7.2	20.0 ± 11.0	0.10
5.7	15.6	26.7 ± 11.0	0.01
5.7	29.4	7.7 ± 11.0	0.50
11.4	1.7	50.0 ± 9.5	<0.001
11.4	7.2	45.3 ± 9.5	<0.001
11.4	15.6	35.0 ± 9.5	0.01
11.4	29.4	17.3 ± 9.5	0.10
22.7	1.7	103.3 ± 9.5	<0.001
22.7	7.2	76.3 ± 9.5	<0.001
22.7	15.6	58.0 ± 9.5	<0.001
22.7	29.4	31.8 ± 9.5	<0.001

^1^ A cow-specific baseline temperature range (BTR) was calculated using the mean ± 2 SD of all reticuloruminal temperature recorded 48 h before the beginning of the study. This BTR was used to define the TIME when reticuloruminal temperature returned to baseline following a drinking event.
